# RMI2 is a novel prognostic and predictive biomarker for breast cancer

**DOI:** 10.1002/cam4.5533

**Published:** 2022-12-19

**Authors:** Lijie Zhang, Chuncheng Hao, Baojuan Han, Guangchun Zeng, Lili Han, Cong Cao, Hui Liu, Zhenbin Zhong, Xue Zhao, Jingxuan Wang, Qingyuan Zhang

**Affiliations:** ^1^ Department of Medical Oncology Harbin Medical University Cancer Hospital Harbin China; ^2^ Department of Head and Neck Radiation Oncology Harbin Medical University Cancer Hospital Harbin China; ^3^ Department of Pathology Harbin Medical University Cancer Hospital Harbin China; ^4^ Department of Orthopedic Surgery, The First Hospital of Suihua Suihua China

**Keywords:** breast cancer, migration, prognosis, proliferation, RMI2

## Abstract

**Background:**

RecQ‐mediated genome instability 2 (RMI2) maintains genome stability by promoting DNA damage repair. It has been reported to accelerate the progression of several tumors. However, the functional mechanism of RMI2 in breast cancer remains unclear.

**Methods:**

Gene expression profiles were obtained from TCGA, GTEx, and GEO databases. The expression of RMI2 and its prognostic value in breast cancer was explored. In addition, we calculated pooled standardized mean deviation (SMD) and performed a summary receiver operating characteristic (sROC) curve analysis to further determine RMI2 expression status and diagnostic significance. The functions and related signaling pathways were investigated based on GO and KEGG analyses. The PPI network was constructed by combining the STRING database and Cytoscape software. Subsequently, in vitro assays were conducted to detect the effect of RMI2 on the proliferation and migration of breast cancer cells.

**Results:**

The expression of RMI2 was markedly upregulated in breast cancer tissues relative to that in normal tissues. Moreover, pooled SMD further confirmed the overexpression of RMI2 in breast cancer (SMD = 1.29, 95% confidence interval (CI): 1.18–1.41, *p* = 0.000). The sROC curve analysis result suggested that RMI2 had a relatively high diagnostic ability in breast cancer (AUC = 0.87, 95% CI: 0.84–0.90). High RMI2 expression was associated with poor prognosis. GO and KEGG analyses revealed that RMI2 was closely related to cell adhesion, various enzyme activities, and PI3K/AKT signaling pathway. PPI analysis showed that RMI2 had interactions with proteins involved in DNA damage repair. knockdown of RMI2 remarkably inhibited the proliferation and migration of breast cancer cells, while overexpression of RMI2 exerted the opposite effects. Furthermore, we identified that RMI2 accelerates the proliferation and migration of breast cancer cells via activation of the PI3K/AKT pathway.

**Conclusion:**

The results suggest that RMI2 is a potential diagnostic and prognostic biomarker associated with cell proliferation and migration, and may be used as a novel therapeutic target for breast cancer in the future.

## Graphical abstract

This study evaluates the differential expression, prognostic value, molecular functions, and related mechanisms of RMI2 in breast cancer.

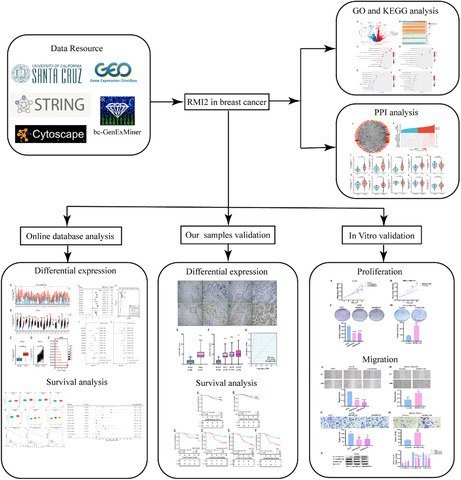

## 1 INTRODUCTION

Breast cancer has surpassed lung cancer as the most common malignancy worldwide with an estimated 2.3 million new cases in 2020, it remained the leading cause of cancer death in females with 0.69 million new deaths.^1^ There are many treatments for breast cancer such as chemotherapy, radiation therapy, endocrine therapy, targeted therapy, and immunotherapy. Although advancements in early detection and treatment modalities have reduced mortality by 38%, there is still a high prevalence of metastasis and recurrence.^2^ Therefore, determining the underlying molecular mechanisms of breast cancer occurrence and development is vital for identifying potential molecular biomarkers and therapeutic targets to improve patient outcomes.

RecQ‐mediated genome instability 2 (RMI2), also known as BALP18, includes an OB‐fold domain. RMI2 is an essential component of the BTR complex (BLM‐Topo IIIα‐RMI1‐RMI2) which can separate the double Holliday junction, a DNA homologous recombination intermediate, and prevent chromosomal aberrations and rearrangements. Therefore, RMI2, as a key member of the BTR complex, plays a vital role in maintaining genome stability.^3^ In addition, RMI2 regulates some important biological processes, such as cell proliferation, invasion and metastasis, cell apoptosis, and cell cycle process, and is closely correlated with the occurrence, development, and prognosis of lung cancer, cervical squamous cell carcinoma (CESC), and hepatocellular carcinoma (HCC). Overexpression of RMI2 accelerated the growth and metastasis of lung cancer and was associated with poor outcomes.^4^, ^5^ Overexpression of RMI2 promoted the growth of HCC cells, facilitated cell cycle phase transition, and suppressed apoptosis by blocking the P53 pathway.^6^ Integrated bioinformatics analysis revealed that RMI2 was overexpressed in CESC, and the expression level was negatively implicated with DNA methylation.^7^ In addition, RMI2 was related to a shorter survival period in pancreatic cancer and could be downregulated by cantharidin.^8^ Because of the limited reports, the role of RMI2 in breast cancer remains unclear.

This study aims to evaluate the aberrant expression, molecular functions, prognostic value, and related mechanisms of RMI2 in breast cancer, and to provide a fundamental for molecular diagnosis and clinical therapy of breast cancer.

## 2 MATERIALS AND METHODS

### 2.1 Data acquisition

Ten microarray datasets related to breast cancer, namely GSE14999, GSE73235, GSE29044, GSE9309, GSE32641, GSE24124, GSE162228, GSE18672, GSE42568, and GSE70947, were screened out from the GEO database (https://www.ncbi.nlm.nih.gov/geo).^9^ The search strategy included the terms: (1) breast cancer samples, (2) homo sapiens, expression profiling by array, and tissues. The inclusion criteria were as followings: (1) more than 100 samples in each dataset, (2) including tumor and normal tissues, and (3) including the expression profile of RMI2. RNA‐seq data of breast cancer tissues from TCGA and normal tissues from GTEx databases as well as clinical information were downloaded from the UCSC Xena (https://xena.ucsc.edu/).

### 2.2 Bioinformatics analysis

Based on the RNA‐seq data of the TCGA‐BRCA samples, differentially expressed genes (DEGs) between RMI2 high and low expression groups, which are defined by the median expression level of RMI2, were identified with the EdgeR package. Adjusted *p*‐value <0.05 was set as the selection criteria. GO and KEGG analyses of the DEGs were performed with the ClusterProfiler package of R. GO analysis mainly covers three areas including the cellular component (CC), molecular function (MF), and biological process (BP). The relevance between RMI2 expression and markers of proliferation (MKI67, PCNA, MCM2, MCM3, MCM4, MCM6) and EMT (β‐catenin, ZO‐1, MMP9) were explored using Pearson analysis.

The bc‐GenExMiner program^10^ (http://bcgenex.ico.unicancer.fr/) was applied to evaluate the relationships of RMI2 expression with clinicopathological factors and prognosis. We applied the STRING (https://string‐db.org/) database^11^ to construct the protein–protein interaction (PPI) network by querying the protein “RMI2” and organism “Homo sapiens” (interaction score >0.7). Then, the network was imported into Cytoscape software (https://cytoscape.org/) for visualization.^12^ The cytoHubba app in Cytoscape was applied to obtain the hub genes in this network using the MCC method. The expression level of hub genes between breast cancer and normal tissues as well as between high and low RMI2 expression groups was analyzed using TCGA data.

### 2.3 Clinical samples

We retrospectively collected 207 breast cancer and 44 adjacent noncancerous breast tissues from patients of Harbin Medical University Cancer Hospital from May 2012 to December 2013. All patients were pathologically confirmed as invasive ductal carcinoma, and patients who underwent anti‐cancer treatments before surgery were excluded. The clinicopathological information of the patients was acquired from the medical records and pathological reports. Follow‐up was performed until December 31, 2020. Seven patients were lost to follow‐up. This study was approved by the ethics committee of Harbin Medical University Cancer Hospital (KY2018‐06).

### 2.4 Immunohistochemistry (IHC)

The tissue slides were deparaffinized at 60°C for 2 h, washed with xylene, and rehydrated using gradient ethanol. 3% Hydrogen peroxide was applied to block the activity of endogenous peroxidases for 10 min. The slides were boiled for 2 min 30 s in sodium citrate buffer to perform antigen retrieval and incubated with anti‐human RMI2 polyclonal antibody (1:200, ab122685, Abcam) at 4 °C overnight, then, incubated with the secondary antibody for 30 min and stained with DAB for 5 min. Each slide was evaluated by two pathologists who selected five representative fields at high magnification (×400). The percentage of positive cells in total tumor cells was scored as 0: <5%, 1: 6%–25%, 2: 26%–50%, 3: 51%–75%, and 4: >75%. The staining intensity of tumor cells was scored as 0: no staining, 1: canary yellow, 2: yellow, and 3: brown. The two scores were multiplied to generate the final IHC score. Positive expression of RMI2 was defined as an IHC score ≥3.^13^

### 2.5 Cell culture and transfection

T47D and MDA‐MB‐231cell lines were acquired from the Heilongjiang Cancer Institute. All cells were cultured in DMEM or L15 medium, both containing 10% fetal bovine serum in an incubator at 37°C. RMI2‐siRNAs and siRNA#NC were designed and purchased from RiboBio (Guangzhou, China). RMI2 overexpression and vector plasmids were acquired from Genechem (Shanghai, China), using a Polyplus transfection reagent.

Cells were plated in 6‐well plates, 18–24 h later, transfected with siRNAs when cell confluence was 30%–50%, or transfected with plasmids when cell confluence was 60%–80%. After 48 h of incubation, cells were used for extracting total RNA and protein. For the mechanism study, MDA‐MB‐231 cells were transfected with overexpression plasmids, 24 h later, LY294002 with a final concentration of 50 μmol/L was added. After 24 h of incubation, total protein was extracted for pathway protein analyses.

The sequences of siRNAs for RMI2 are as follows:

siRMI2#1: GACAGACCTTTCTGATAAT.

siRMI2#2: GTGGGAACTGGAGGTAGAA.

siRMI2#3: TGGTGATGGGAGTGGTTCA.

### 2.6 Quantitative real‐time PCR

Total RNA was extracted using TRIzol. 1 μg RNA was reverse transcribed into cDNA using the RT kit (KR118). The SYBR Green SuperReal PreMix Plus kit (FP205) was applied for real‐time PCR. The relative expression level was estimated using the 2^−ΔΔCT^ method. GAPDH was used for normalization. Specific primer sequences for RMI2, BLM, BRCA2, FANCA, FANCD2, FANCM, PALB2, RMI1, TOP3A, RPA1, RPA2, and GAPDH are listed in Table S2.

### 2.7 Western blot

Total protein was extracted using RIPA lysis buffer containing PMSF (volume ratio 99:1) and phosphatase inhibitors (volume ratio 100:1). Protein concentration was estimated using a BCA kit. Total protein (20–40 μg) was separated by 10% or 15% SDS‐PAGE, then transferred to 0.2 μm PVDF membranes (Millipore). After blocking with 5% defatted milk for 1 h, the PVDF membranes were probed with the specific primary antibodies overnight at 4°C, then probed with the secondary antibody for 1 h at room temperature. The protein bounds were detected using the ECL Western Blotting Detection system. The antibodies information is listed in Table S3.

### 2.8 Cell proliferation assays

For the CCK‐8 assay, cells were plated in 96‐well plates (4 × 10^3^/well) and cultured for some specific time points (24, 48, 72, and 96 h). To each well, the 100 μl mixture solution of CCK8 and medium (volume ratio 1:9) was added. After incubation for 3 h. The absorbance value (450 nm) was detected. To examine the colony formation capacity, cells (500 cells/well) were plated in 6‐well plates and cultured for about 10–14 days. The cells were fixed and stained when visible clones formed.

### 2.9 Cell migration assays

For the wound healing assay, transfected cells were cultured in 6‐well plates until the cells covered the whole hole. Then, we made a scratch with a 200 μl pipette tip and continued to culture the cells in the serum‐free medium for 48 h. Images were taken at 0 and 48 h. The migration index defined as the healing area/initial gap area was measured using the ImageJ software. Transwell assay was applied in an 8um chamber (Corning). Transfected cells (T47D, 7 × 10^4^/well; MDA‐MB‐231, 4 × 10^4^/well) were plated in the top chamber, and the medium with 30% FBS was plated in the lower wells. Twenty‐four h later, cells in the upper chamber were removed, and migratory cells were fixed and stained.

### 2.10 Statistical analysis

GraphPad Prism 8.0, SPSS 20.0, and R (version 3.6.3) software were applied for statistical analyses. Comparisons between groups were conducted using *t*‐test and ANOVA. Associations between RMI2 expression and clinicopathologic factors were examined by the Chi‐square test. Survival outcomes between high and low RMI2 expression groups were compared using the log‐rank test. The prognostic values of factors for OS were evaluated using Cox regression models; univariate analysis was conducted first, and factors with a *p*‐value <0.05 were then examined using multivariate analysis. STATA 17.0 was used to undertake the meta‐analysis. The standard mean deviation (SMD) was adopted to confirm RMI2 expression in breast cancer and normal samples. The heterogeneity of 11 breast cancer datasets (TCGA‐BRCA and 10 GEO microarrays) was estimated via a heterogeneity test, with *I* ^2^ < 50% signifying no heterogeneity. A random effect model may be conducted if heterogeneity existed. To further identify the diagnostic value of RMI2 in breast cancer, a summary receiver operating characteristics (sROC) curve was plotted and the area under the curve (AUC) was calculated. *p* < 0.05 indicates statistically significant.

## 3 RESULTS

### 3.1 Increased RMI2 expression in public databases

**FIGURE 1:**
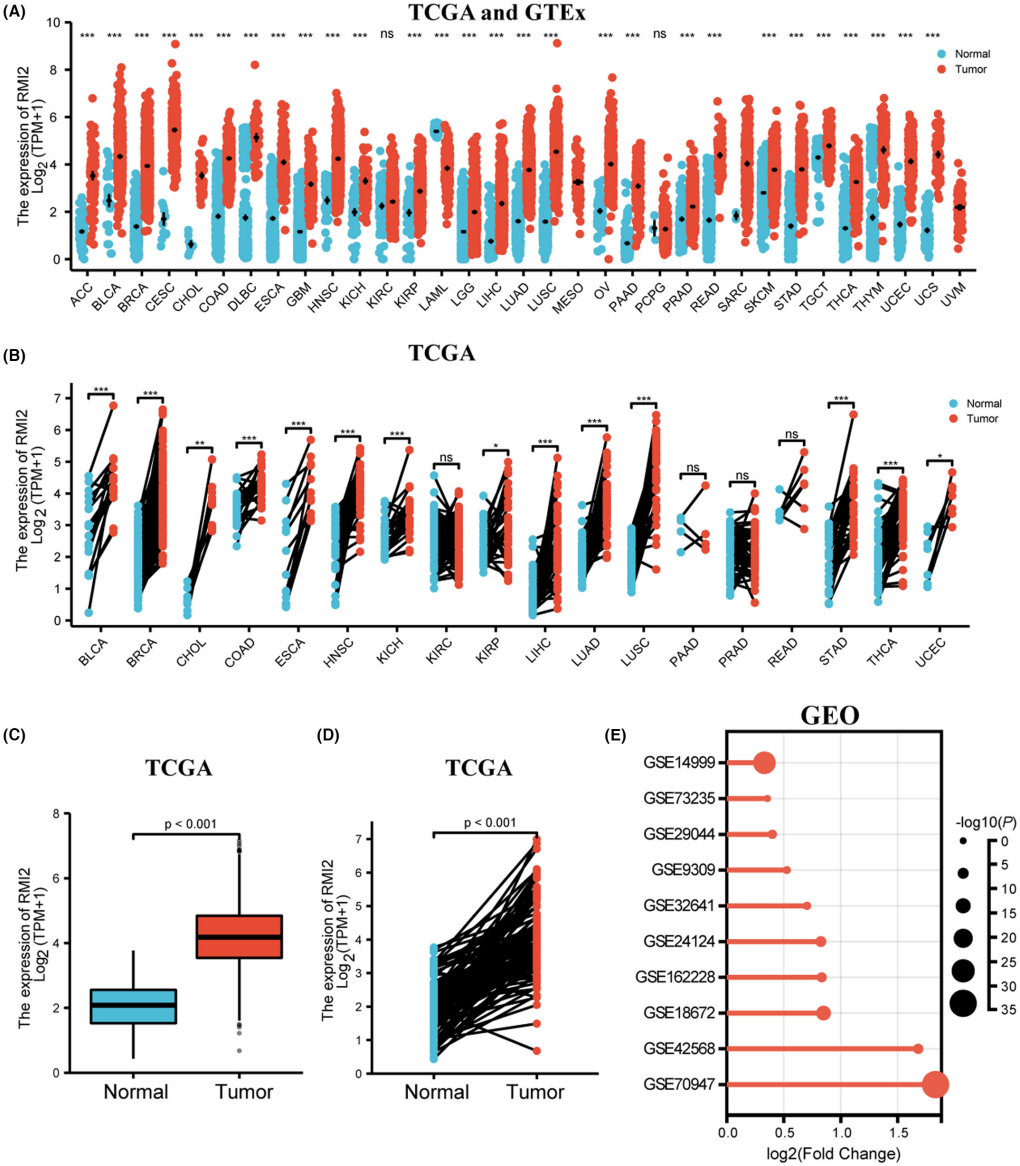
The expression levels of RMI2 in public databases. (A) Expression of RMI2 in different types of tumors in TCGA and GTEx databases. (B) Expression of RMI2 in paired tumor and normal tissues in the TCGA database. Expression levels of RMI2 in nonpaired breast cancer tissues and normal tissues (C) as well as paired samples (D). (E) RMI2 was upregulated in breast cancer tissues compared to normal tissues in multiple datasets except GSE73235 in the GEO database. The ordinate is the datasets, the abscissa is log2 (Fold Change), and the point size represents significance (indicated by the inverse log10 of *p* values). *p < 0.05, **p < 0.01, ***p < 0.001.

To examine RMI2 expression in different types of tumors, we downloaded the data of TCGA and GTEx and used R language for data processing and analysis. Results showed that when compared with normal tissues, RMI2 mRNA was highly expressed in multiple tumor tissues (Figure 1A,B). Subsequently, the expression pattern of RMI2 in breast cancer and normal tissues was further explored. The result from TCGA showed that RMI2 was overexpressed in nonpaired (Figure 1C) and paired (Figure 1D) samples. In addition, the high expression of RMI2 in breast cancer tissues was further confirmed in GSE14999 (*p* = 1.44 E‐26), GSE29044 (*p* = 8.82 E‐05), GSE9309 (*p* = 0.016241), GSE32641 (*p* = 0.003768), GSE24124 (*p* = 1.90 E‐07), GSE162228 (*p* = 3.32 E‐06), GSE18672 (*p* = 8.52 E‐14), GSE42568 (*p* = 2.00 E‐06), and GSE70947 (*p* = 2.90 E‐34), however except GSE73235 (*p* = 0.29888) (Figure 1E).

**FIGURE 2:**
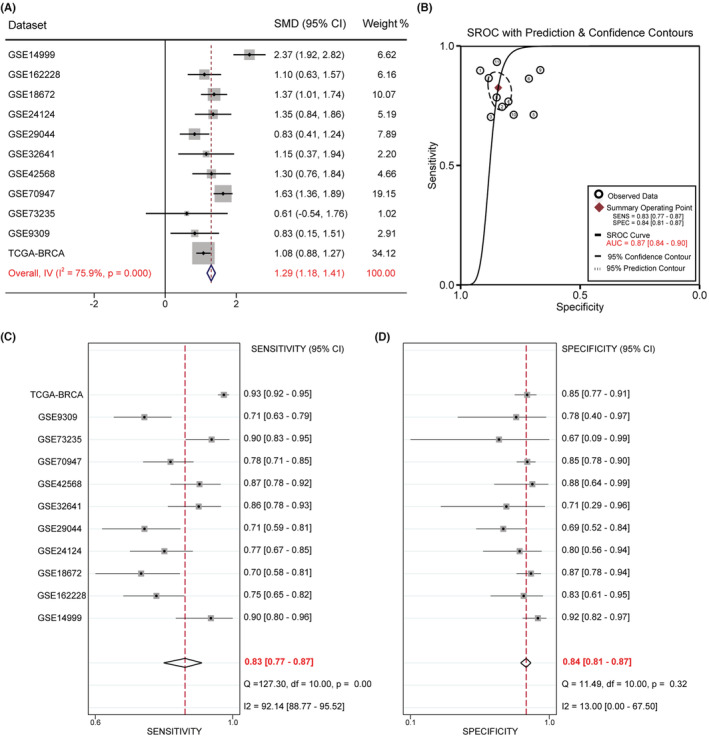
Comprehensive analysis of RMI2 expression level in breast cancer based on 11 datasets from TCGA and GEO databases. (A) Forest plot of studies evaluating standard mean deviation (SMD) of RMI2 mRNA expression between breast cancer tissues and normal tissues. Because of the high heterogeneity (*I* ^2^ = 75.9%, *p* = 0.000), a random effect model was used. SMD for each dataset is represented by the square, and the horizontal line crossing the square represents the 95% confidence interval (CI). The diamond represents the estimated overall effect. (B) Summary receiver operating characteristic (sROC) curve for the diagnostic accuracy assessment of RMI2 in breast cancer. The sensitivity (C) and specificity (D) values of the included studies.

We further integrated TCGA RNA‐seq and 10 GEO microarray data for meta‐analysis. The main information of the datasets is shown in Table S1. This research included 2088 cancer tissues and 513 normal tissues. Considering the large heterogeneity (*I* ^2^ = 75.9%, *p* = 0.000) (Figure 2A), we adopted a random effect model to calculate the SMD and 95% confidence interval (CI). The pooled SMD of RMI2 was 1.29 (95% CI: 1.18–1.41, *p* = 0.000) (Figure 2A), which indicated that RMI2 expression was significantly upregulated in breast cancer tissues compared to normal tissues. Finally, sROC analysis was carried out to determine the diagnostic ability of RMI2 in breast cancer. Independent ROC curves of each dataset are presented in Figure S1. The overall combined AUC was 0.87 (95% CI: 0.84–0.90) (Figure 2B), with a combined sensitivity of 0.83 (95% CI: 0.77–0.87) (Figure 2C) and a combined specificity of 0.84 (95% CI: 0.81–0.87) (Figure 2D), thus indicating an upper‐middle diagnostic ability of RMI2 for breast cancer.

### 3.2 Associations between RMI2 expression and clinicopathological factors and prognosis

**FIGURE 3:**
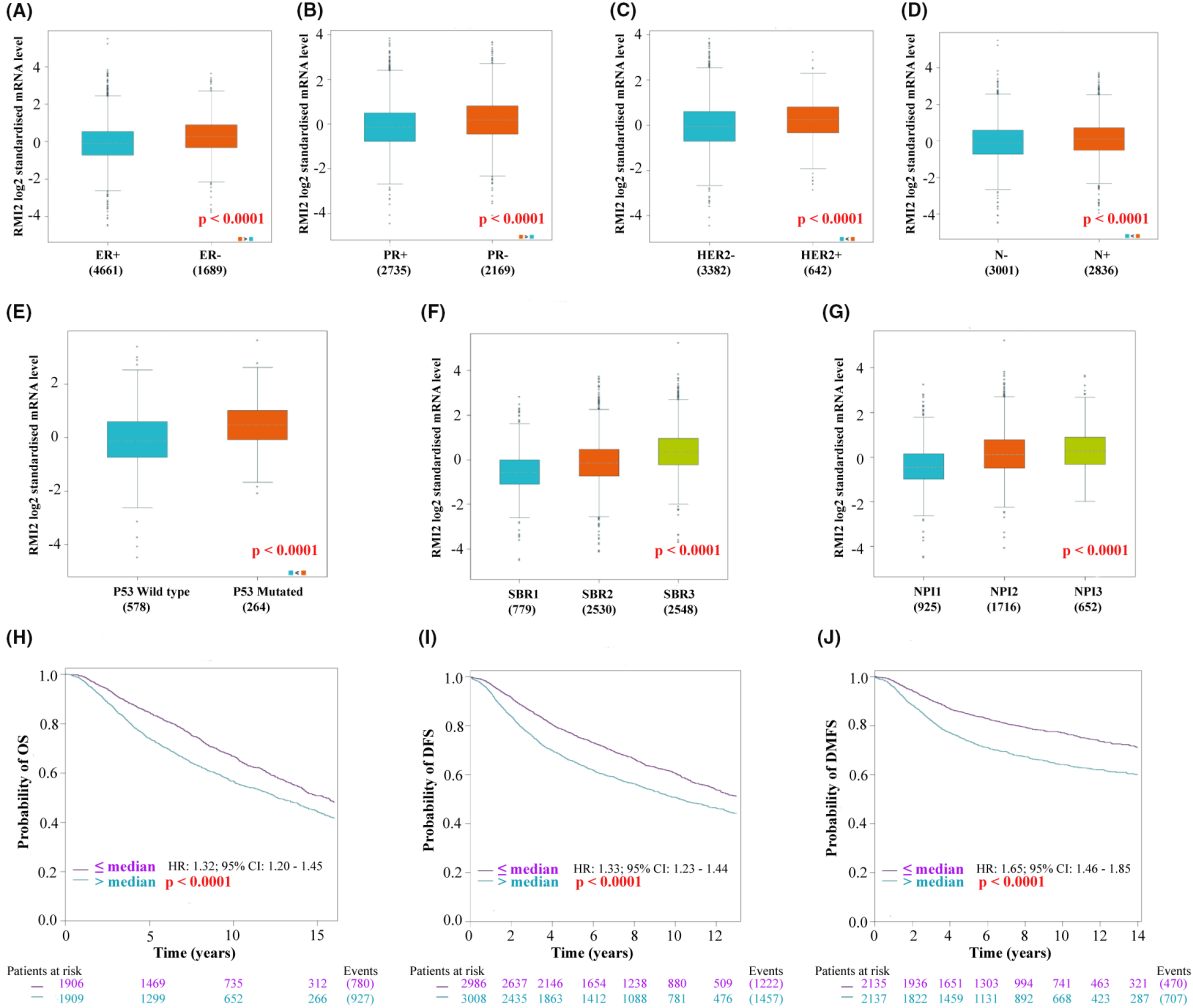
Associations of RMI2 expression with clinicopathological factors, and survival in bc‐GenExMiner database. The Box plot shows the association between RMI2 expression and ER status (A), PR status (B), HER2 status (C), nodal status (D), p53 status (E), Scarff‐Bloom‐Richardson grade (SBR grade) (F), Nottingham Prognostic Index (NPI) (G). Kaplan–Meier survival analysis of the relationship between RMI2 expression and overall survival (OS) (H), disease‐free survival (DFS) (I), and distant metastasis‐free survival (DMFS) (J).

The correlations of RMI2 expression with multiple clinicopathological factors were examined through the bc‐GenExMiner database, We found that RMI2 was upregulated in ER‐negative (Figure 3A), PR‐negative (Figure 3B), and HER2‐positive (Figure 3C) groups. The expression of RMI2 was elevated in the lymph node metastasis group (Figure 3D). Milner et al.^14^ stated that the mutant p53 gene losses the functions of tumor suppression and promotes the malignant transformation of cells and tumor progression. As expected, in the p53‐mutated group, the expression of RMI2 was significantly increased (Figure 3E). The Scarff‐Bloom‐Richardson grade (SBR grade) is evaluated based on tube formation, nuclear pleomorphism, and mitotic index.^15^ The Nottingham Prognostic Index (NPI) is evaluated by tumor size, tumor grade, and lymph node stage.^16^ These two methods are mainly used for risk stratification of breast cancer patients and are considered to be very useful prognostic models. Results suggested that RMI2 expression was increased in patients with high SBR grade and NPI (Figure 3F,G), indicating that elevated RMI2 expression predicts poor clinical outcomes. As shown in Figure S2, there were significant differences in the expression of RMI2 among different subtypes of breast cancer, and the expression of RMI2 was relatively higher in the basal‐like and luminal B types based on the TCGA database.

**FIGURE 4:**
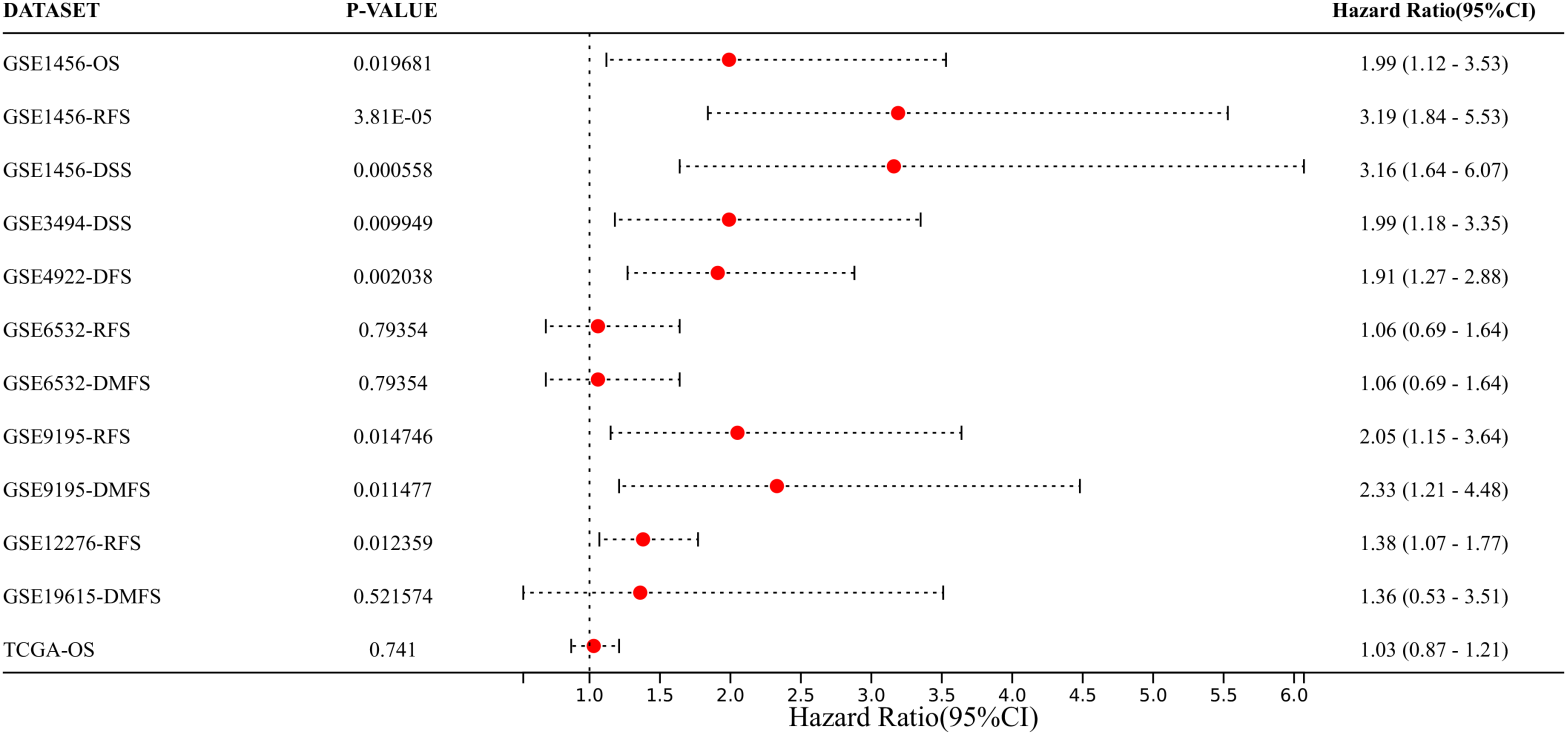
Forest plot of the relationship between RMI2 expression and overall survival (OS), relapse‐free survival (RFS), disease‐specific survival (DSS), disease‐free survival (DFS), and distant metastasis‐free survival (DMFS) in breast cancer patients. CI, confidence interval.

Subsequently, we analyzed the influence of RMI2 on overall survival (OS), disease‐free survival (DFS), and distant metastasis‐free survival (DMFS) to confirm its prognostic significance for breast cancer patients. Results suggested that breast cancer patients with higher expression of RMI2 had shorter OS, DFS, and DMFS (Figure 3H–J). To further verify the prognostic value of RMI2, we used multiple datasets (GSE1456, GSE3494, GSE4922, GSE6532, GSE9195, GSE12276, GSE19615, TCGA) and clinical outcome status (OS; DFS; DMFS; relapse‐free survival, RFS; disease‐specific survival, DSS). The forest plot revealed that high RMI2 expression was significantly associated with poor prognosis (HR >1) (Figure 4).

### 3.3 Potential mechanisms of RMI2 in regulating the progression of breast cancer

**FIGURE 5:**
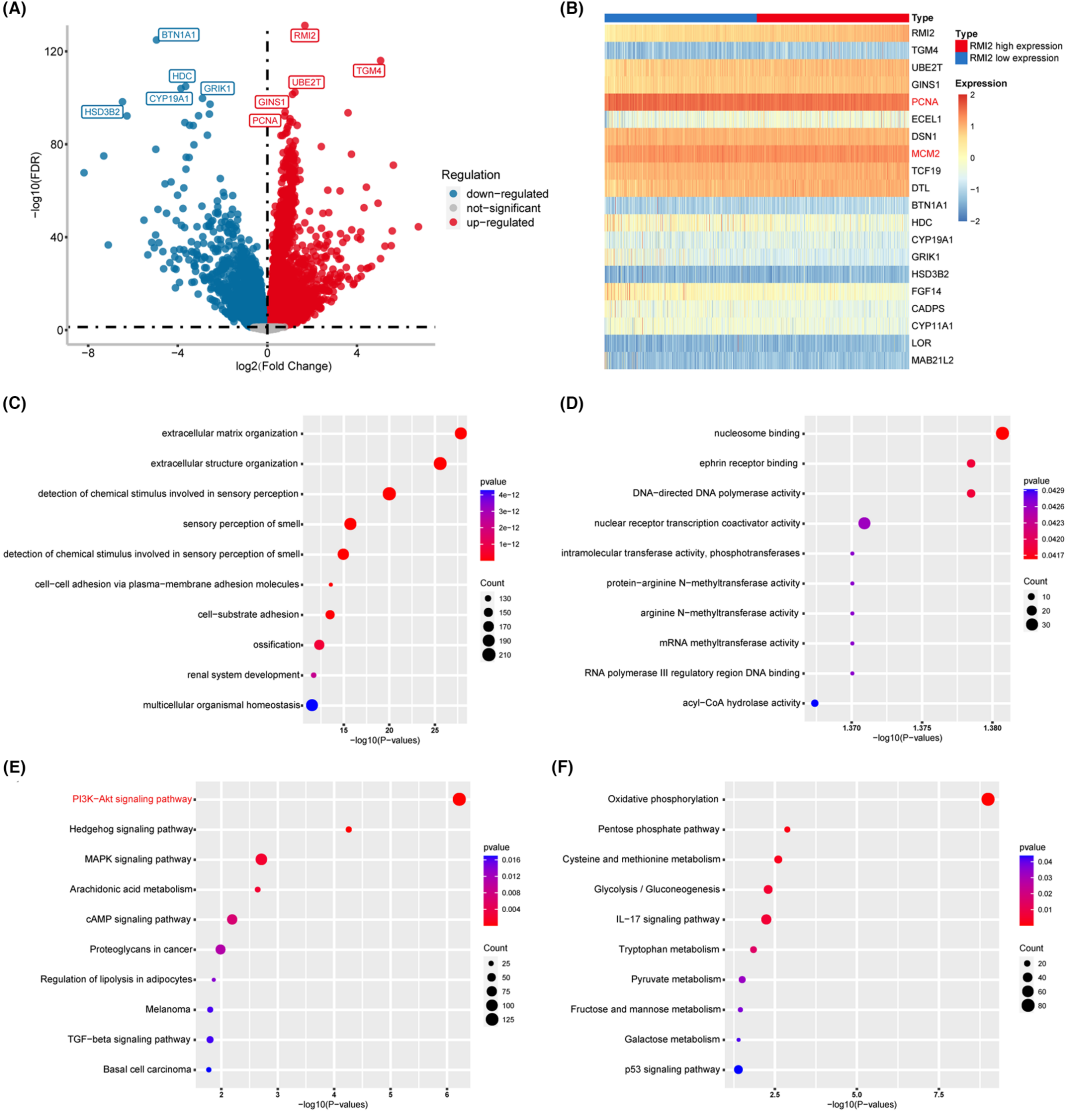
Functional annotation and predictive signaling pathways. (A) A volcano plot of the differentially expressed genes (DEGs) between high and low RMI2 expression groups. Globally DEGs (red means up‐regulated and blue means down‐regulated) are defined as adjusted *p*‐value <0.05. (B) Heat map showing the expression of the top 19 DEGs with RMI2 high and RMI2 low expression. (C, D) Top 10 significantly enriched gene ontology (GO) terms in RMI2 low (C) and RMI2 high groups (D). (E, F) Top 10 significantly enriched KEGG pathways in RMI2 low (E) and RMI2 high (F) groups.

The EdgeR package was utilized to calculate DEGs between two groups. Among these genes, 7074 genes were up‐regulated in the RMI2 high expression group, and 5669 genes were upregulated in the RMI2 low expression group (Figure 5A). Additionally, markers associated with proliferation such as PCNA and MCM2 were significantly differentially expressed (Figure 5B). Subsequently, the DEGs were subject to GO and KEGG analyses. The GO items encompassed multiple functions such as cell adhesion, nucleosome binding, DNA polymerase activity, phosphotransferase activity, methyltransferase activity, and hydrolase activity (Figure 5C,D). Results of KEGG analysis showed that the DEGs were mainly enriched in PI3K/AKT, Hedgehog, MAPK, as well as metabolism‐associated signaling pathways such as glycolysis, pyruvate metabolism, and tryptophan metabolism (Figure 5E,F).

**FIGURE 6:**
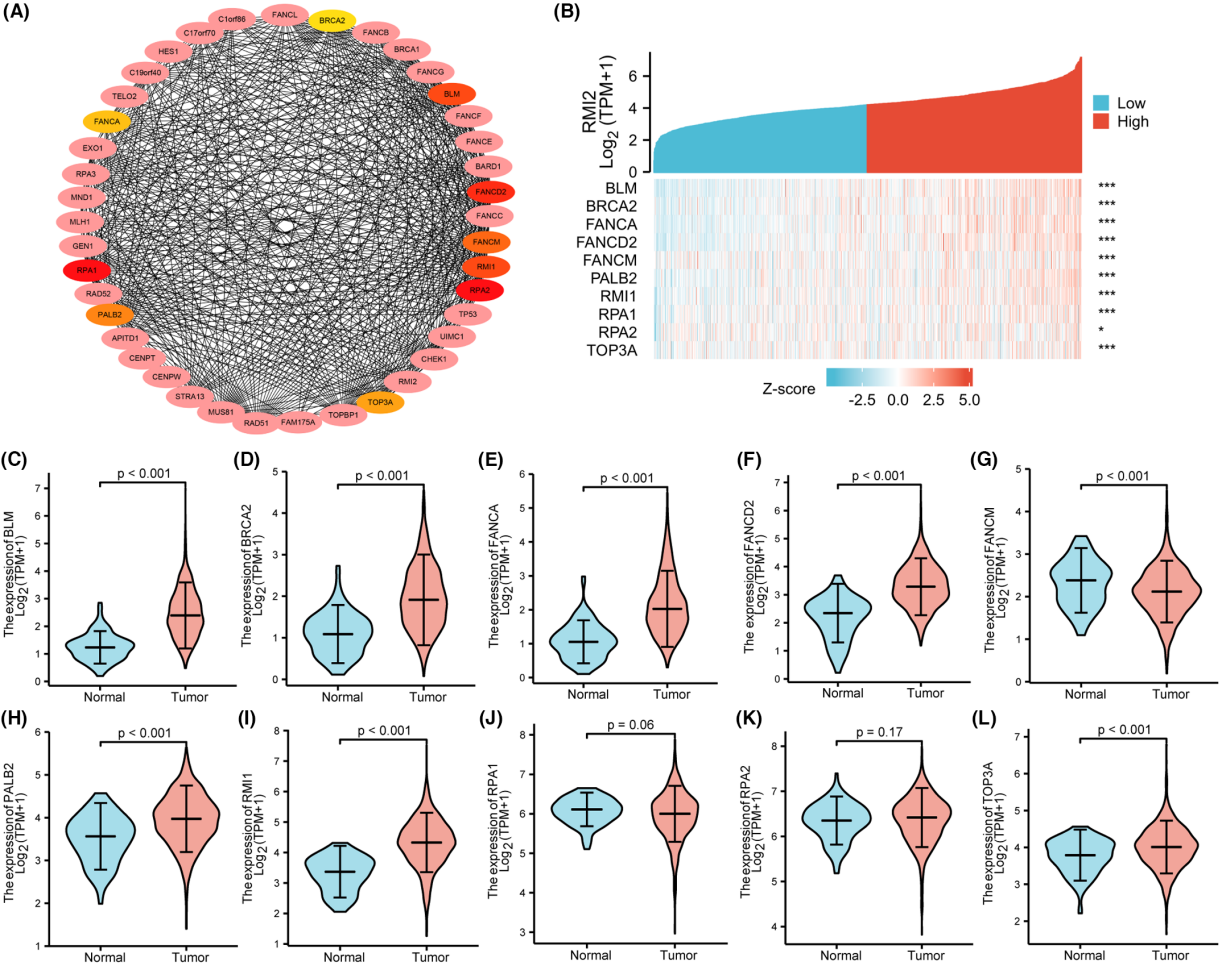
(A) The protein–protein interaction network of RMI2 was constructed based on the STRING database (interaction score >0.7) and Cytoscape software. A total of 10 hub genes were obtained from the cytoHubba app in Cytoscape using the MCC algorithm. (B) Heatmap of the expression of hub genes with RMI2 high and RMI2 low expression. The asterisk character represents the *p*‐value of the statistical result of the correlation between hub genes and RMI2. *p < 0.05; ***p < 0.001. (C–L) Violin plots showing the expression of hub genes in breast cancer tissues and normal tissues from the TCGA database.

The hub genes in the PPI network of RMI2 were BLM, FANCD2, RPA1, RPA2, FANCM, RMI1, PALB2, FANCA, TOP3A, and BRCA2 (Figure 6A) which are involved in DNA damage repair process. The co‐expression heatmap of RMI2 with these genes is shown in Figure 6B. These genes were markedly differentially expressed in breast cancer tissues compared to normal tissues except for RPA1 and RPA2 (Figure 6C–L). In addition, based on the median value of RMI2 expression, breast cancer samples from TCGA were divided into high and low RMI2 expression groups. Most of the hub genes in the high RMI2 expression group were markedly upregulated compared with the low RMI2 expression group (Figure S3A). Moreover, qRT‐PCR results confirmed that BRCA2 (*p* = 0.0003), FANCA (*p* = 0.0007), FANCD2 (*p* < 0.0001), FANCM (*p* = 0.0003), RPA1 (*p* = 0.0001), and TOP3A (*p* = 0.0125) were significantly decreased in the RMI2 knockdown group (Figure S3B–K).

**FIGURE 7:**
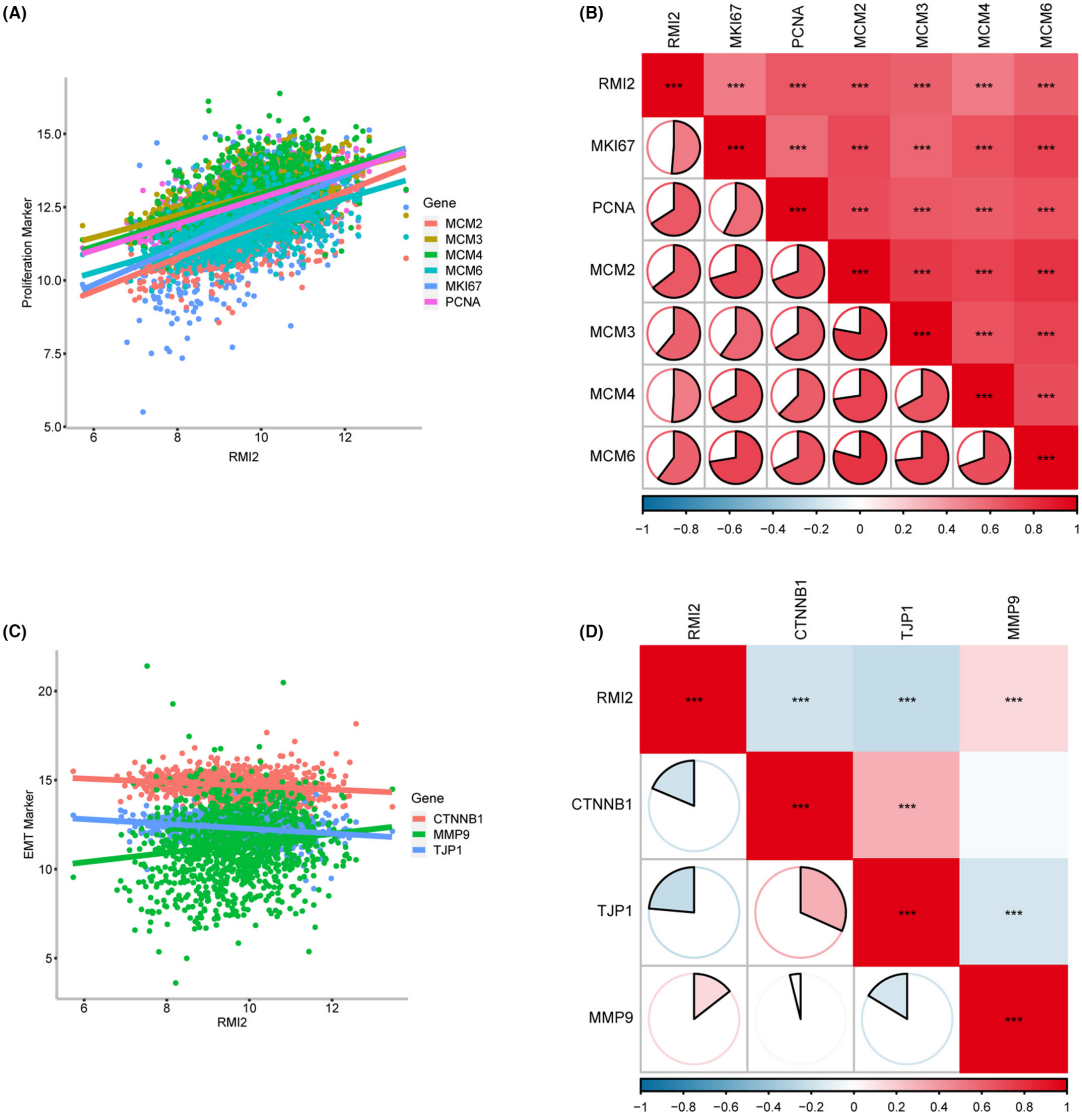
Relationships between RMI2 and proliferation and EMT in breast cancer samples from TCGA. (A, B) Pearson's correlation between RMI2 and proliferation‐related genes, including MKI67 (*r* = 0.51), PCNA (*r* = 0.66), MCM2 (*r* = 0.64), MCM3 (*r* = 0.61), MCM4 (*r* = 0.51), and MCM6 (*r* = 0.60). (C, D) Pearson's correlation between RMI2 and EMT‐related genes including CTNNB1 (*r* = −0.19), TJP1 (*r* = −0.24), and MMP9 (*r* = 0.14). ***p < 0.001.

Since proliferation and EMT were considered the main features of cancer, we explored the associations of RMI2 with markers of proliferation and EMT. Results showed that RMI2 was positively correlated with MKI67, PCNA, MCM2, MCM3, MCM4, MCM6, and MMP9, however, negatively correlated with CTNNB1 and TJP1 which were encoding genes of epithelial markers β‐catenin, and ZO‐1 (Figure 7).

### 3.4 RMI2 protein was upregulated in our clinical breast cancer samples

**FIGURE 8:**
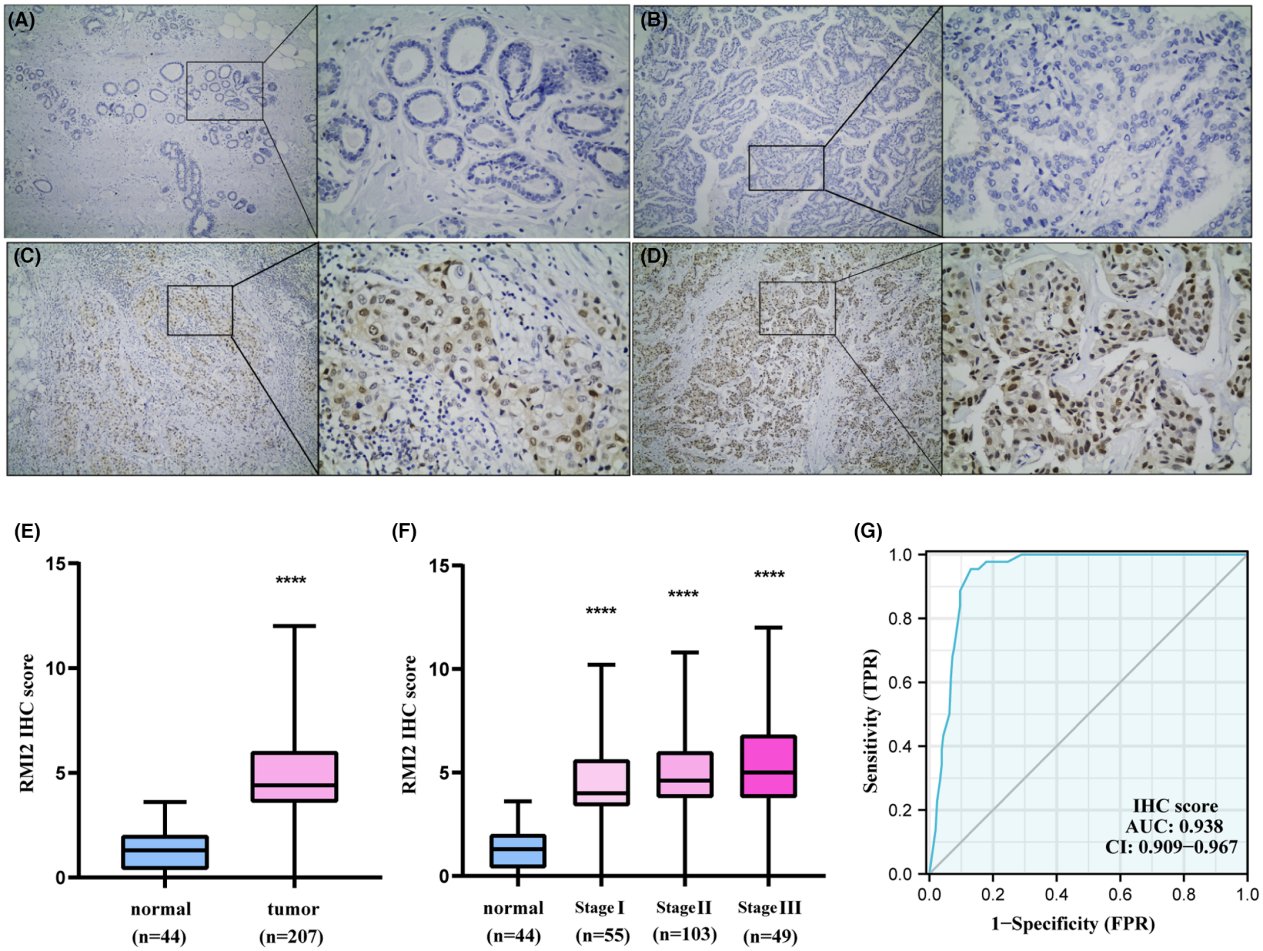
RMI2 protein expression in our clinical breast cancer patients. (A) Negative RMI2 expression in normal tissue (×100, ×400). (B) Negative RMI2 expression in breast cancer tissue (×100, ×400). (C) Moderate RMI2 expression in breast cancer tissue (×100, ×400). (D) Strongly positive RMI2 expression in breast cancer tissue (×100, ×400). (E) The expression of RMI2 protein in breast cancer tissues was higher than that in normal tissues (*p* < 0.0001). (F) The expression of RMI2 protein in stages I‐III was statistically significant compared with the normal group in our study (all *p* < 0.0001). (G) ROC curve showing the diagnostic value of RMI2 for breast cancer.

To verify the findings obtained from the bioinformatics databases. We performed the IHC assay to detect RMI2 protein expression. The detailed IHC score information is shown in Table S4. 85.5% (177/207) of primary breast cancer patients were RMI2 positive expression. Consistent with the results of the bioinformatics database, RMI2 protein levels were increased in breast cancer tissues (Figure 8A–E). We also found that RMI2 expression in stage I‐III disease was increased compared with the normal group, and when the stage increased, the expression of RMI2 was slightly elevated (Figure 8F).

### 3.5 Clinical significance of RMI2 in our breast cancer samples

**FIGURE 9:**
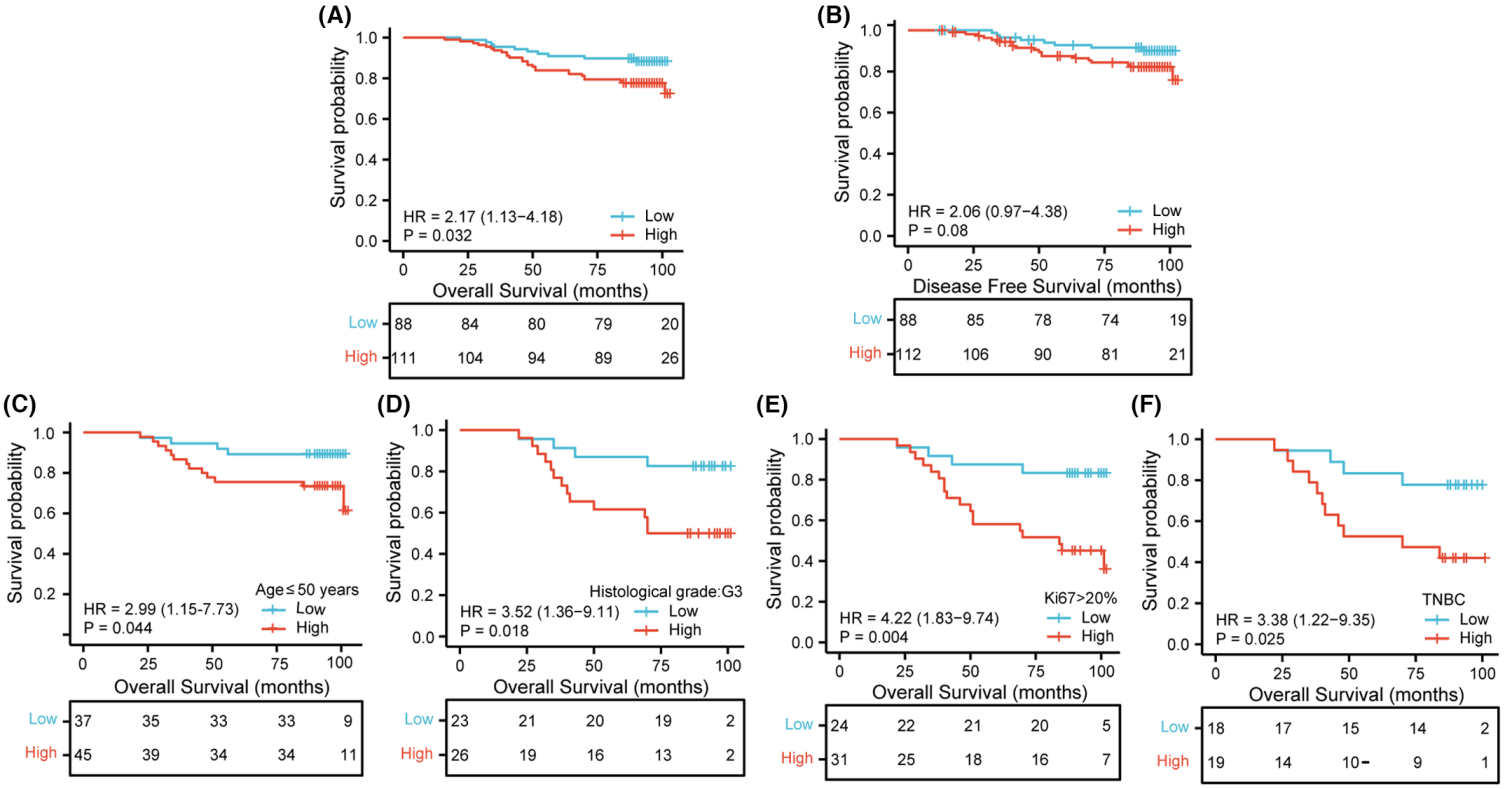
Prognostic value of RMI2 in our clinical breast cancer specimens. (A, B) Associations between RMI2 expression and overall survival (OS), disease‐free survival (DFS). Associations between RMI2 expression and OS in breast cancer subgroups, including age ≤ 50 years (C), histological grade G3 (D), Ki67 > 20% (E), TNBC subtype (F).

ROC analysis was performed using our clinical specimens. The result revealed that the AUC of RMI2 was 0.938 (Figure 8G). Using the median IHC score, 207 patients were divided into high and low RMI2 expression groups. The Chi‐square test revealed that RMI2 protein levels were significantly correlated with the pT (*p* = 0.020) and pTNM stage (*p* = 0.049), but were not correlated with other parameters (Table 1). Subsequently, we collected the survival data of 200 patients to assess the associations between RMI2 expression and survival time. Results suggested that patients in the high RMI2 expression group had shorter OS (*p* = 0.032, Figure 9A) and DFS (*p* = 0.08, Figure 9B). However, DFS did not reach a statistical difference. Subgroups analyses, including age ≤50 years (*p* = 0.044, Figure 9C), G3 grade (*p* = 0.018, Figure 9D), Ki67 > 20% (*p* = 0.004, Figure 9E), and TNBC group (*p* = 0.025, Figure 9F) revealed that the survival curves diverged more significantly, indicating that the prognostic value of RMI2 in some aggressive subgroups was more pronounced. Then, we explored the risk factors affecting patient prognosis. Univariate analysis revealed that histological grade (HR = 3.393, 95% CI: 1.764–6.525, *p* = 0.000), pLN metastasis (HR = 1.766, 95% CI: 1.255–2.486, *p* = 0.001), pTNM stage (HR = 1.773, 95% CI: 1.094–2.873, *p* = 0.020), ER status (HR = 0.336, 95% CI: 0.173–0.652, *p* = 0.001), PR status (HR = 0.388, 95% CI: 0.197–0.767, *p* = 0.006), Ki67 status (HR = 4.997, 95% CI: 2.553–9.779, *p* = 0.000), molecular subtype (HR = 1.873, 95% CI: 1.408–2.491, *p* = 0.000), and RMI2 expression (HR = 2.170, 95% CI: 1.046–4.501, *p* = 0.037) were risk parameters for poor prognosis (Table 2). Furthermore, multivariate analysis of these eight factors showed that pLN metastasis (HR = 2.259, 95% CI: 1.191–4.285, *p* = 0.013), molecular subtype (HR = 1.776, 95% CI: 1.013–3.113, *p* = 0.045), and RMI2 expression (HR = 2.528, 95% CI: 1.165–5.486, *p* = 0.019) were independent risk factors for poor prognosis of breast cancer (Table 2). These findings suggested the potential clinical worth of RMI2 in breast cancer.

**TABLE 1:** Relationships between RMI2 expression and clinicopathologic characteristics of breast cancer

Characteristics	Number (207)	RMI2		*p*‐value
		Low expression (90)	High expression (117)	
Age (years)				
≤50	83	37	46	0.794
>50	124	53	71	
Histological grade				
G1 + G2	157	67	90	0.632
G3	50	23	27	
pT				
pT1	104	52	52	**0.020**
pT2	96	38	58	
pT3	7	0	7	
pLN metastasis				
No	97	49	48	0.107
Yes	110	41	69	
pTNM stage				
I	55	30	25	**0.049**
II	103	45	58	
III	49	15	34	
Menopause				
Before	78	34	44	0.980
After	129	56	73	
ER status				
Negative	73	32	41	0.939
Positive	134	58	76	
PR status				
Negative	90	39	51	0.971
Positive	117	51	66	
HER2 status				
Negative	155	72	83	0.136
Positive	52	18	34	
Ki67 status				
≤20%	149	65	84	0.946
>20%	58	25	33	
P53 status				
Negative	149	68	81	0.315
Positive	58	22	36	

*Note*: *p* values in bold means statistically significant.

**TABLE 2:** Cox regression analyses for overall survival

	Univariate analysis			Multivariate analysis		
Variable	HR	95% CI	*p*	HR	95% CI	*p*
Age (≤50 vs. >50)	0.735	0.382–1.414	0.356	—	—	—
Histological grade	3.393	1.764–6.525	**0.000**	1.803	0.818–3.974	0.144
pT stage	0.978	0.529–1.808	0.944	—	—	—
pLN metastasis	1.766	1.255–2.486	**0.001**	2.259	1.191–4.285	**0.013**
pTNM stage	1.773	1.094–2.873	**0.020**	0.556	0.216–1.436	0.225
Menopause status	0.757	0.392–1.460	0.406	—	—	—
ER status	0.336	0.173–0.652	**0.001**	1.082	0.223–5.244	0.922
PR status	0.388	0.197–0.767	**0.006**	1.1454	0.310–6.822	0.635
HER‐2 status	1.164	0.561–2.416	0.683	—	—	—
Ki67 (≤20% vs. >20%)	4.997	2.553–9.779	**0.000**	2.036	0.873–4.748	0.100
P53 status	1.938	0.991–3.788	0.053	—	—	—
Molecular subtype	1.873	1.408–2.491	**0.000**	1.776	1.013–3.113	**0.045**
RMI2 expression	2.170	1.046–4.501	**0.037**	2.528	1.165–5.486	**0.019**

*Note*: *p* values in bold means statistically significant.

Abbreviations: HR, hazard ratio; CI, confidence interval.

### 3.6 The roles of RMI2 knockdown and overexpression on tumorigenicity of breast cancer

**FIGURE 10:**
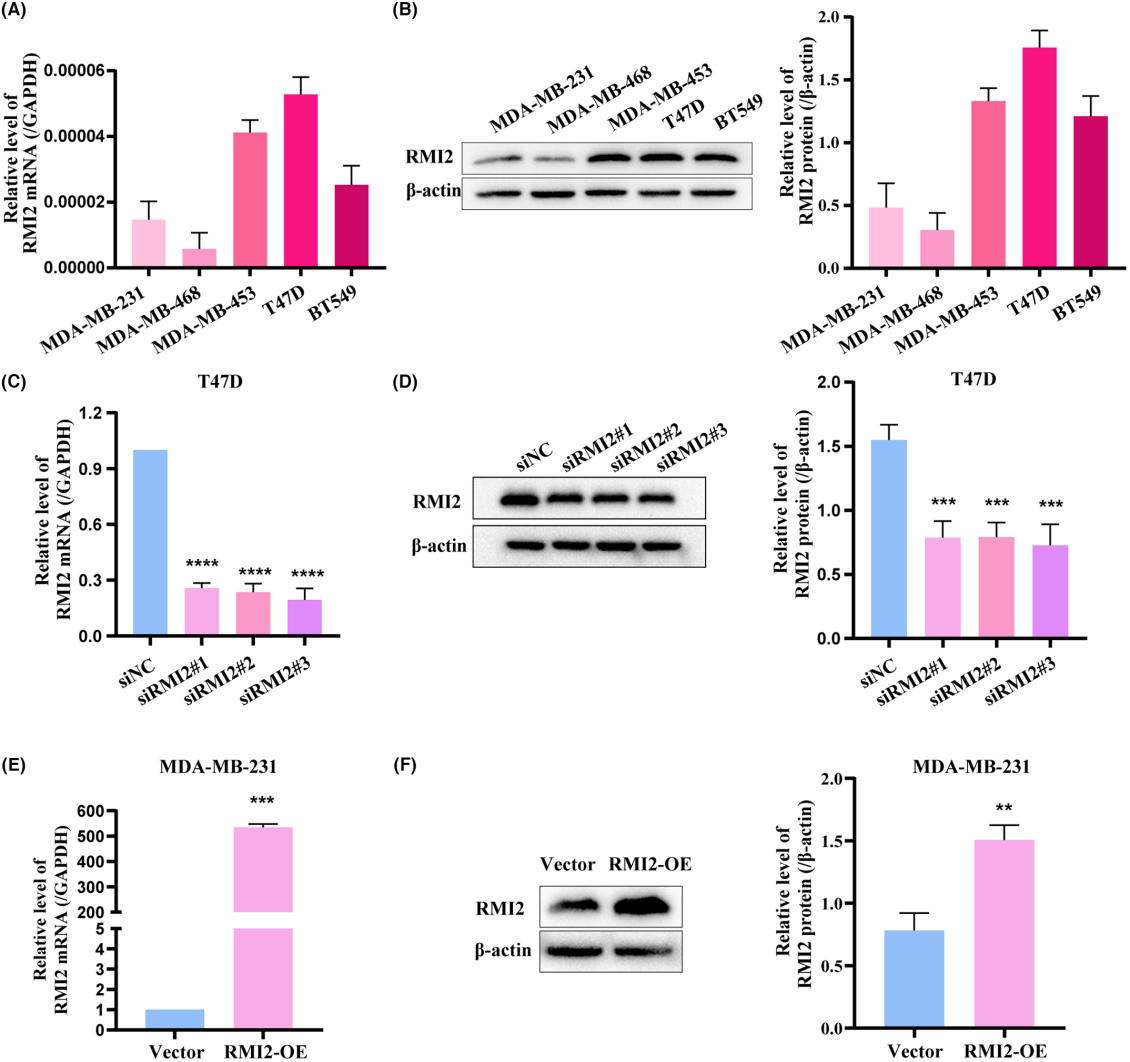
The expression levels of RMI2 in breast cancer cell lines. (A) qRT‐PCR showed the relative mRNA levels of RMI2. (B) Western blot showed protein levels of RMI2. (C) qRT‐PCR showed mRNA levels of RMI2 in T47D cells transfected with siNC or RMI2 siRNAs. (D) Western blot showed knockdown of RMI2 in T47D cells. (E) qRT‐PCR showed mRNA levels of RMI2 in MDA‐MB‐231 cells transfected with overexpression plasmids. (F) Western blot showed overexpression of RMI2 in MDA‐MB‐231 cells. Data were shown as mean ± SD. **p < 0.01, ***p < 0.001, ****p < 0.0001.

Subsequently, the mRNA and protein levels of RMI2 in different breast cancer cell lines were investigated. Results showed relatively high expression of RMI2 in T47D cells and relatively low expression in MDA‐MB‐231 cells (Figure 10A,B). RMI2 siRNA was transfected into T47D cells. Meanwhile, the MDA‐MB‐231 cell line with RMI2 overexpression was established by transfection with overexpressed plasmids. Transfection efficiency was tested by qRT‐PCR and western blot (Figure 10C–F). The interference efficiency of the three siRNAs was basically the same. However, the interference of siRMI2#2 was unstable. Then siRMI2#1 and siRMI2#3 were selected for subsequent assays.

**FIGURE 11:**
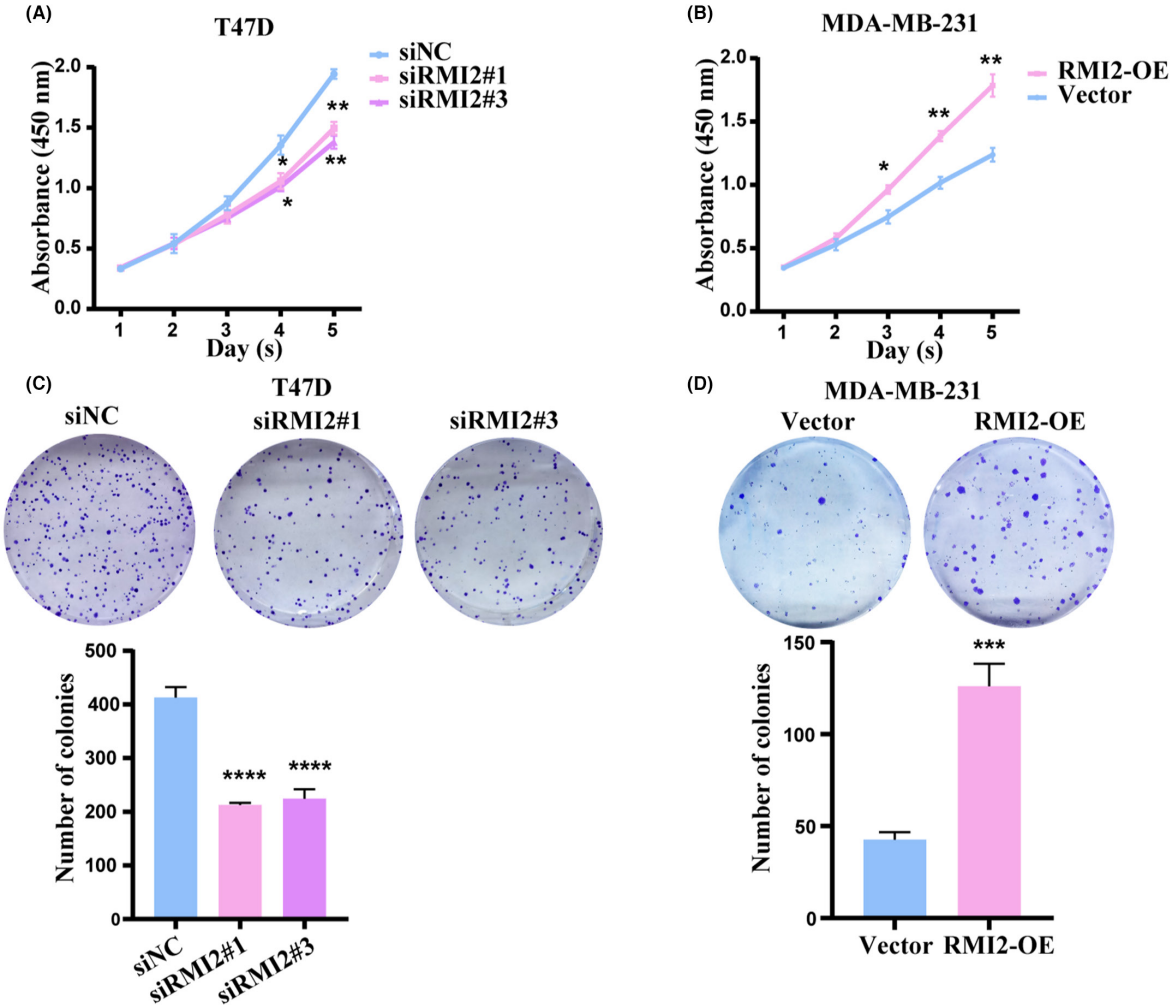
RMI2 promotes the proliferation ability of breast cancer cells. CCK‐8 assay showed the cell proliferation ability of T47D cells with RMI2 knockdown (A) and MDA‐MB‐231 cells with RMI2 overexpression (B). Colony formation ability of T47D cells with RMI2 knockdown (C) and MDA‐MB‐231 cells with RMI2 overexpression (D). Data were presented as means ± SD. *p < 0.05, **p < 0.01, ***p < 0.001, ****p < 0.0001.

**FIGURE 12:**
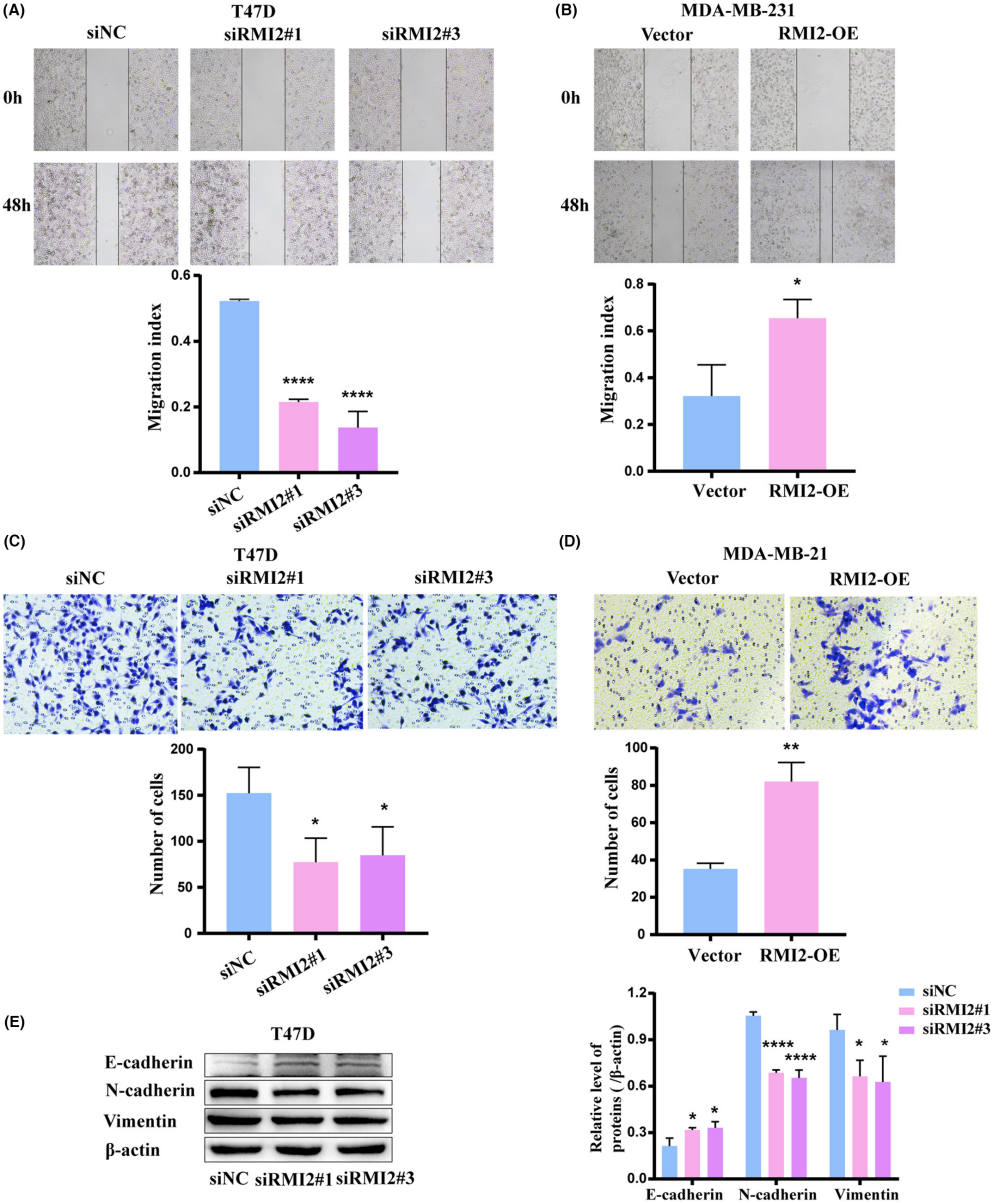
Effect of RMI2 on cell migration ability of breast cancer cells. Wound healing assays showed the migration capacity of T47D cells with RMI2 knockdown (A) and MDA‐MB‐231 cells with RMI2 overexpression (B). Transwell assays showed the migration capacity of T47D cells with RMI2 knockdown (C) and MDA‐MB‐231 cells with RMI2 overexpression (D). (E) Western blot of EMT markers in siNC, siRMI2#1, and siRMI2#3 groups. Data were presented as means ± SD. *p < 0.05, **p < 0.01, ****p < 0.0001.

CCK‐8 assays and colony formation assays showed that RMI2 knockdown significantly inhibited cell proliferation and colony formation ability in T47D cells (Figure 11A,C). Additionally, wound healing assays and transwell assays demonstrated that the migration ability of T47D cells in the siRMI2 group was suppressed compared with the siNC group (Figure 12A,C). The functions of RMI2 were further explored by performing overexpression assays. Results revealed that when compared with the vector group, elevated expression of RMI2 remarkably enhanced cell proliferation and migration abilities (Figure 11B,D, 12B,D). In addition, we used western blot to examine the protein expression of EMT markers. As shown in Figure 12E, in the siRMI2 group, the expression of E‐cadherin was increased, whereas the expression of N‐cadherin and Vimentin was decreased.

### 3.7 RMI2 promoted cell proliferation and migration by the PI3K‐AKT pathway

**FIGURE 13:**
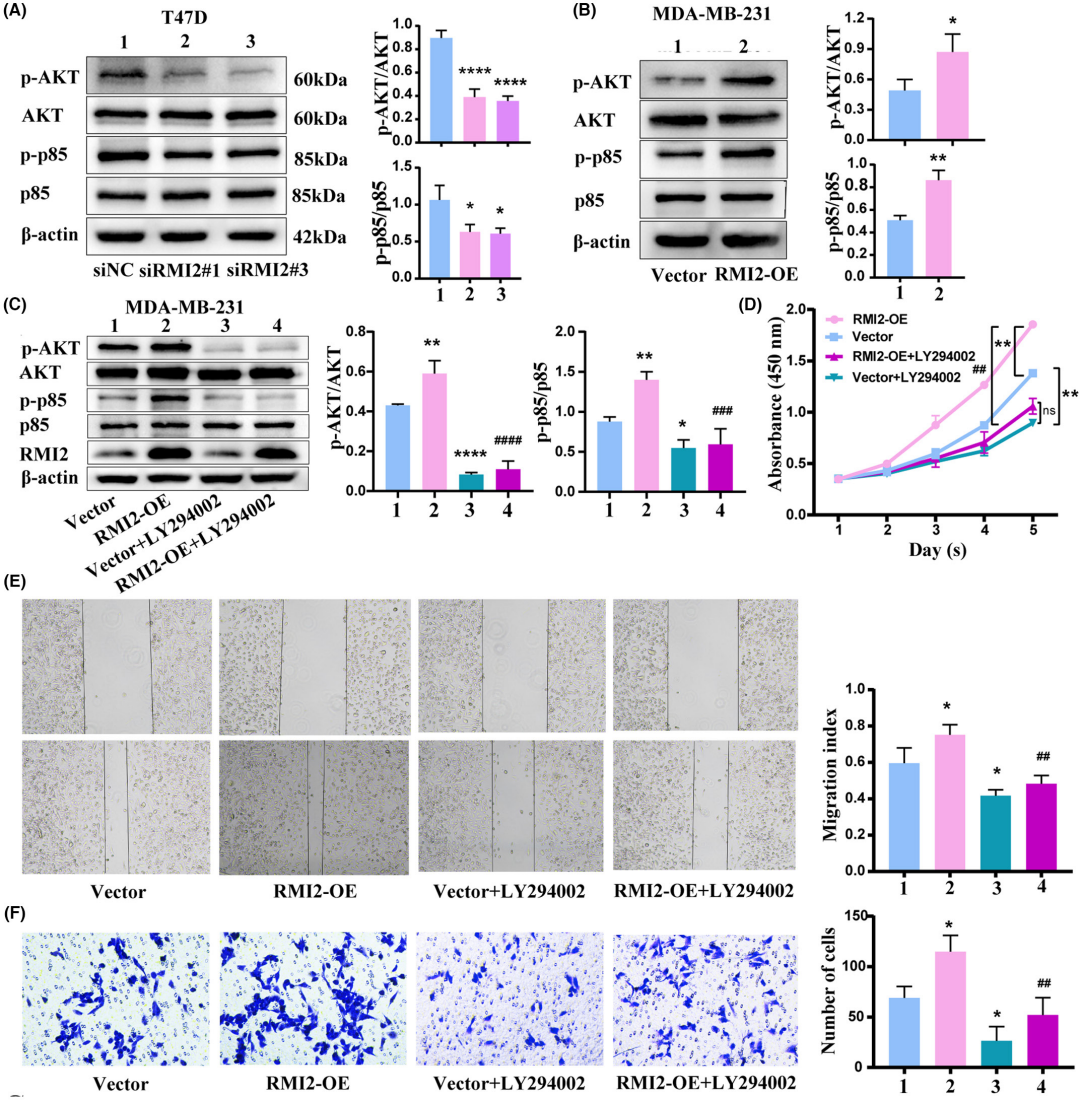
RMI2 promotes breast cancer progression by activating the PI3K‐AKT signaling pathway. The protein levels of p‐AKT, AKT, p‐p85, and p85 were detected by western blot in T47D cells with RMI2 knockdown (A) and MDA‐MB‐231 cells with RMI2 overexpression (B). MDA‐MB‐231 cells transfected with RMI2 overexpression plasmids, 24 h later, treated with LY294002, detecting p‐AKT, AKT, p‐p85, p85 expression by Western blot (C), detecting cell viability by CCK‐8 assay (D), evaluating cell migration capability by wound healing and Transwell assays (E, F). Data were presented as means ± SD. * versus Vector group, # versus RMI2‐OE group. *p < 0.05, **p < 0.01, ****p < 0.0001, ##*p* < 0.01, ###*p* < 0.001, ####*p* < 0.0001.

Bioinformatics analysis revealed that DEGs were enriched in PI3K/AKT, Hedgehog, MAPK, and metabolism‐associated signaling pathways. Via western blot, we observed that the protein levels of p‐AKT and p‐p85 in the RMI2 knockdown cells were markedly reduced, which were elevated in the RMI2 overexpressed cells, with total AKT and p85 expression unchanged (Figure 13A,B). Subsequently, LY294002 (a PI3K inhibitor) was used to explore whether RMI2 mediated breast cancer progression through PI3K/AKT pathway. After the addition of LY294002, the expression of p‐Akt and p‐p85 was dramatically decreased, while total AKT and p85 were unchanged (Figure 13C). In function terms, CCK‐8 assays demonstrated that the increase of cell viability mediated by RMI2 overexpression was reversed by LY294002 (Figure 13D). Similarly, it was also revealed that LY294002 blocked the enhancement of the migrative capability of MDA‐MB‐231 cells mediated by RMI2 overexpression (Figure 13E,F). These results revealed that RMI2 facilitated cell proliferation and migration through the PI3K‐AKT pathway.

## 4 DISCUSSION

Breast cancer is the most common malignancy worldwide. Despite considerable progress in treatment achieved in recent years, curing this disease remains a challenge, particularly in advanced cases.^17^ To improve disease cure rates and patient survival, the identification of various molecular biomarkers for early diagnosis and prognosis assessment is imperative. RMI2 is essential for maintaining the stability of the genome. Some reports have revealed that RMI2 is closely associated with a variety of cancers and is considered to be an oncogene. Loss of RMI2 results in genomic instability and can cause Bloom‐like syndrome.^18^ Because of the limited number of reports, the function of RMI2 in breast cancer remains unclear.

Here, we first performed an integrated analysis of the expression data of 2601 samples from different datasets to calculate SMD and sROC. This study on RMI2 expression in breast cancer had the largest sample size of any related study by far. Results revealed that RMI2 was markedly overexpressed in breast cancer tissues. In addition, RMI2 was correlated with multiple clinicopathological factors and poor prognosis. However, the results of the bc‐GenexMiner database are not completely consistent with the IHC assay. This discrepancy may be caused by the following reasons such as the heterogeneity of the population, selection bias, our small sample size, different IHC scoring methods, and different positive thresholds.

The biological functions of RMI2 in cancer have rarely been studied. Zhan et al.^4^ reported that RMI2 shuttles from the cytoplasm to the nucleus by forming the BTR complex to facilitate cell proliferation and tumor growth of lung cancer, it also promotes tumor metastasis by upregulating SLUG. Li et al.^6^ found that RMI2 could promote the proliferation of HCC cells and inhibit their apoptosis. Zhang et al.^19^ reported that RMI2 knockdown remarkably inhibited the proliferation, colony formation, and migration of prostate cancer cells. Subsequently, we explored whether RMI2 affected malignant phenotypes of breast cancer. A series of functional experiments demonstrated that RMI2 accelerated breast cancer cell proliferation and migration, and these phenotypic changes were consistent with the above studies.

In the KEGG analysis of RMI2, it was found to be related to multiple cancer‐related signaling pathways in breast cancer, such as PI3K/AKT, Hedgehog, and MAPK signaling pathways. PI3K/AKT is a star pathway in the development of tumors. PI3Ks are lipid kinases, which can be divided into three categories.^20^ The most widely studied is class I PI3K mainly containing regulatory subunit p85 and catalytic subunit p110.^21^ PI3Ks phosphorylate PIP2 to PIP3, which further recruits and activates AKT.^22^ The PI3K/AKT pathway is abnormally activated in multiple cancers.^23^ It is an important signaling pathway involved in tumorigenesis, proliferation, apoptosis, EMT, metastasis, microenvironment, metabolism, and treatment resistance.^24^, ^25^ At present, this pathway has become an important target for cancer therapy, and many inhibitors targeting this pathway have been tested in preclinical or clinical trials.^26^ In addition, RMI2 was observed to regulate various metabolism processes, such as glycolysis, pyruvate metabolism, and tryptophan metabolism. Recently, metabolism reprogramming especially aerobic glycolysis is considered a hallmark of cancer.^27^, ^28^ Accumulated evidence showed the importance of glycolysis in cancer cell proliferation and metastasis.^29^ Thus, glycolysis has become an attractive target for cancer treatment.^30^

Via PPI analysis and qRT‐PCR verification, we found the proteins that closely interacted with RMI2 were BRCA2, FANCA, FANCM, FANCD2, RPA1, and TOP3A. Most of them are related to the susceptibility of hereditary cancers, and mutation or inactivation of these genes can impair genomic stability and promote tumorigenesis.^31^, ^32^ Among them, the functions of BRCA2 are the most important and deeply studied. BRCA2 is a tumor suppressor gene that plays a crucial role in homologous recombination (HR) repair.^33^ Deficiency in BRCA1/2 induces profound cellular sensitivity to poly (ADP‐ribose) polymerase (PARP) inhibitors.^34^ However, for different tumor types, the therapeutic effects of PARP inhibitors vary greatly, with a response rate of 30%–60%.^35^ Moreover, the remission period is not durable, and drug resistance inevitably occurs.^36^ These factors limit the application of PARP inhibitors. Maybe rational combination strategies can resolve these problems. Li et al.^37^ reported that enzalutamide induces HR deficiency by suppressing the expression of BRCA1, RAD54L, and RMI2 in prostate cancer, thereby, enhancing the therapeutic effect of olaparib. Hence, pharmaceutically inducing HR deficiency may expand the clinical application of PARP inhibitors. Kumar et al.^38^ stated that PI3K signaling pathway preserves the steady state of HR and PI3K inhibition cause HR deficiency which can increase the sensitivity to PARP inhibitors. Ibrahim et al.^39^ reported that PI3K inhibition reduces the expression of BRCA 1/2, leads to HR deficiency, and sensitizes to PARP inhibition. Multiple preclinical studies have shown these synergistic antitumor activities of dual blocking of PI3K and PARP in breast cancer, prostate cancer, and ovarian cancer regardless of PIK3CA or BRCA mutational status, which broadens the indications of PARP inhibitors.^40^, ^41^, ^42^

EMT reduces intercellular adhesion and enhances cell motility which would lead to metastasis of tumor cells.^43^ Therefore, EMT plays an essential role in tumor progression.^44^ In addition, EMT is related to tumor initiation, resistance to therapy, tumor stemness, vascular invasion, and immune evasion.^43^, ^45^, ^46^ The complex biological process of EMT is considered a pivotal feature of cancer, and targeting the EMT pathway is a very attractive strategy for cancer therapy. Functional analysis suggested the close correlation of RMI2 with cell adhesion. We also demonstrated the promotion role of RMI2 in EMT through western blot. A recent study showed that RMI2 was significantly correlated with tumor mutation burden, microsatellite instability, immune cell infiltration, immune checkpoints, and mismatch repair‐related genes^47^ which suggests that RMI2 may influence the response to immunotherapy.

Our research helps clarify the correlations between RMI2 and breast cancer. This is the first study to explore the associations of RMI2 with the proliferation and migration of breast cancer cells, as well as the PI3K/AKT signaling pathway. Moreover, we combined 11 datasets to analyze the expression of RMI2 in breast cancer. Datasets from different sources and a large sample size ensured the credibility of our research. However, some limitations also existed in our study. First, all samples used in this study were collected retrospectively. Therefore, inner tumor heterogeneity and selection bias might affect the results. Second, the findings need to be validated in animal experiments and prospective clinical trials. Finally, the functional mechanism of RMI2 affecting the progression of breast cancer needs further exploration.

## 5 CONCLUSION

In summary, our study suggested that RMI2 is markedly upregulated in breast cancer and its high expression is correlated with poor prognosis. In addition, in vitro assays demonstrated that RMI2 accelerates the proliferation and migration of breast cancer cells by activating the PI3K/AKT pathway.

## AUTHOR CONTRIBUTIONS

**Lijie Zhang:** Data curation (lead); investigation (equal); methodology (equal); validation (lead); visualization (lead); writing – original draft (lead); writing – review and editing (lead). **Chuncheng Hao:** Data curation (equal); validation (equal). **Baojuan Han:** Data curation (equal); writing – original draft (equal). **Guangchun Zeng:** Methodology (equal). **Lili Han:** Software (equal); visualization (equal). **Cong Cao:** Data curation (equal); visualization (equal). **Hui Liu:** Data curation (equal); visualization (equal). **Zhenbin Zhong:** Data curation (equal); methodology (equal); visualization (equal). **Xue Zhao:** Data curation (equal); investigation (equal). **Jingxuan Wang:** Conceptualization (lead); funding acquisition (lead); methodology (lead); resources (lead); supervision (lead). **Qingyuan Zhang:** Conceptualization (lead); methodology (lead); resources (lead); supervision (lead).

## FUNDING INFORMATION

The research was supported by the National Natural Science Foundation of China (Grant No. 81472490 and 81772813).

## CONFLICT OF INTEREST

The authors declare no conflict of interest.

## Supporting information


Data S1.

## Data Availability

RNA‐seq data and corresponding clinicopathologic information on breast cancer were downloaded from UCSC Xena (https://xena.ucsc.edu/) platform. The data of GSE14999, GSE73235, GSE29044, GSE9309, GSE32641, GSE24124, GSE162228, GSE18672, GSE42568, and GSE70947 were downloaded from the GEO database (https://www.ncbi.nlm.nih.gov/geo/).
