# Supplementary material for: RMI2 is a novel prognostic and predictive biomarker for breast cancer
Source: Cancer Med. 2022 Dec 19;12(7):8331–50. doi: 10.1002/cam4.5533 (PMC10134310; doi:10.1002/cam4.5533)
Supplement: Supplementary file 1 — Data S1. [file CAM4-12-8331-s001.zip › CAM4_5533_LijiezhangTableS1-3.docx]

Table S1 Overview of the datasets used for meta-analysis

| **Datasets** | **Author** | **Year** | **Platform** | **Normal** | **Tumor** |
| --- | --- | --- | --- | --- | --- |
| GSE14999 | Uva P | 2009 | GPL3991 | 61 | 68 |
| GSE162228 | Chen Y | 2021 | GPL570 | 23 | 110 |
| GSE18672 | Helland | 2010 | GPL6848 | 79 | 64 |
| GSE24124 | Liu LD | 2010 | GPL887 | 20 | 99 |
| GSE29044 | Colak D | 2014 | GPL570 | 36 | 73 |
| GSE32641 | Liu LD | 2012 | GPL887 | 7 | 95 |
| GSE42568 | Clarke C | 2013 | GPL570 | 17 | 104 |
| GSE70947 | Quigley DA | 2016 | [GPL13607](https://www.ncbi.nlm.nih.gov/geo/query/acc.cgi?acc=GPL13607) | 146 | 144 |
| GSE73235 | Gao Q | 2016 | GPL10558 | 3 | 109 |
| GSE9309 | Yao HC | 2007 | [GPL887](https://www.ncbi.nlm.nih.gov/geo/query/acc.cgi?acc=GPL887) | 9 | 132 |
| TCGA | NCI | 2006 | Gencode v22 | 112 | 1090 |

NCI: National Cancer Institute

Table S2 Primers sequences used in this work

| **Gene** | **Forward Primer (5'-3')** | **Reverse Primer (5'-3')** |
| --- | --- | --- |
| RMI2 | ATGCAGGGCAGGGTAGTGAT | CTTTCCTGGGACTAGACAGGG |
| BLM | CAGACTCCGAAGGAAGTTGTATG | TTTGGGGTGGTGTAACAAATGAT |
| BRCA2 | CAGGTAGACAGCAGCAAGCA | AAGCCCCTAAACCCCACTTC |
| FANCA | GCTTGAGGTAGAAGGTCCACT | CCTGCAAAGCAGAGCCTATAAAT |
| FANCD2 | AAAACGGGAGAGAGTCAGAATCA | ACGCTCACAAGACAAAAGGCA |
| FANCM | CTTAATTGGCGCTGGAATCC | CCACTAAAGACAGTTCCTTTAGCAAA |
| PALB2 | CTCACACAAATATCAGCACGAAAA | GGAATCCTCTTTTTGATGACGACT |
| RMI1 | GTGCGATCCTCAAGAGCGTA | CAGACATCCATCAGCCGGAC |
| TOP3A | ACTTTGGAACGAGAGACTCGC | GGGCTTTACAGCCTTACACAC |
| RPA1 | CCAGCCTCACTCCTTACCAG | TTCACCACTTTCGTCAACCA |
| RPA2 | TTAAGATCATGCCCCTGGAG | ATAGGTGCTCTCCCTGCTGA |
| GAPDH | TGACTTCAACAGCGACACCC | CTGGTGGTCCAGGGGTCTTA |

Table S3 Information of antibodies used in the study

| **Antibodies** | **Manufacturer** | **Catalog Number** | **Dilution** | **Usage** |
| --- | --- | --- | --- | --- |
| RMI2 | Abcam | 122685 | 1:1000 | WB |
| RMI2 | Abcam | 122685 | 1:200 | IHC |
| AKT | CST | 4685 | 1:1000 | WB |
| p-AKT | CST | 9271 | 1:1000 | WB |
| p85 | Proteintech | 60225-1-Ig | 1:1000 | WB |
| p-p85 | Abmart | T40065 | 1:1000 | WB |
| β-actin | Proteintech | 66009-1-Ig | 1:10000 | WB |
| E-cadherin | Proteintech | 20874-1-AP | 1:1000 | WB |
| N-cadherin | Proteintech | 22018-1-AP | 1:5000 | WB |
| Vimentin | Proteintech | 10366-1-AP | 1:5000 | WB |
