# Supplementary figures and images for: RMI2 is a novel prognostic and predictive biomarker for breast cancer
Source: Cancer Med. 2022 Dec 19;12(7):8331–50. doi: 10.1002/cam4.5533 (PMC10134310; doi:10.1002/cam4.5533)

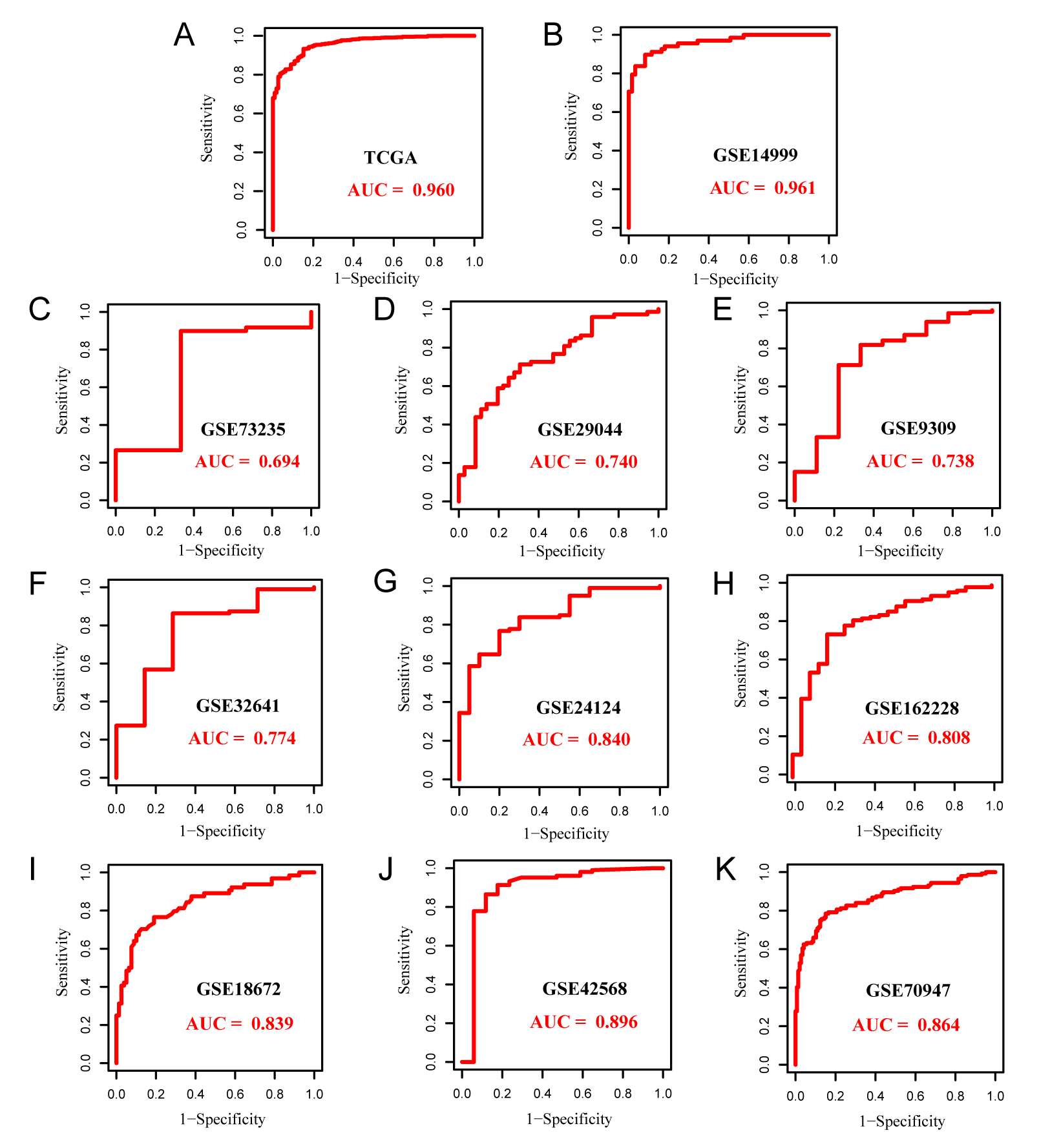

Supplement: Supplementary file 1 — Data S1. [file CAM4-12-8331-s001.zip › CAM4_5533_LijiezhangFigureS1.tif]

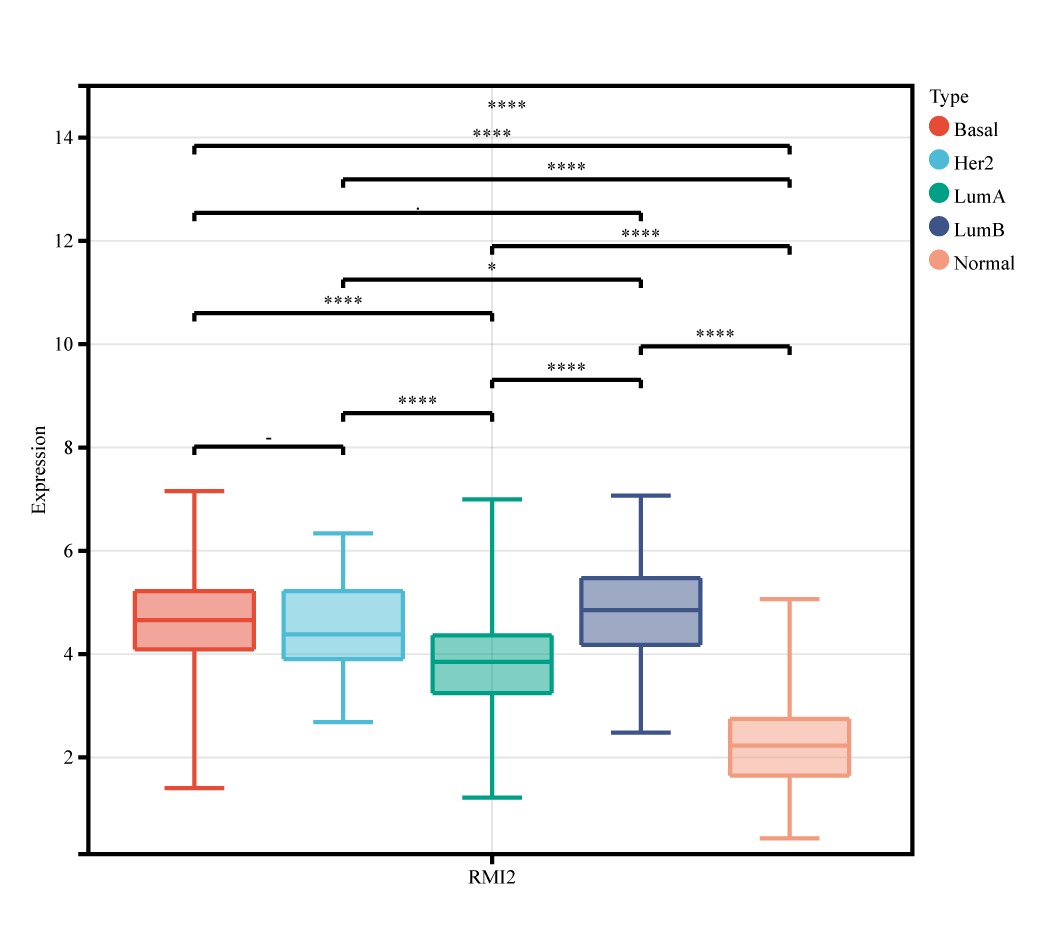

Supplement: Supplementary file 1 — Data S1. [file CAM4-12-8331-s001.zip › CAM4_5533_LijiezhangFigureS2.tif]

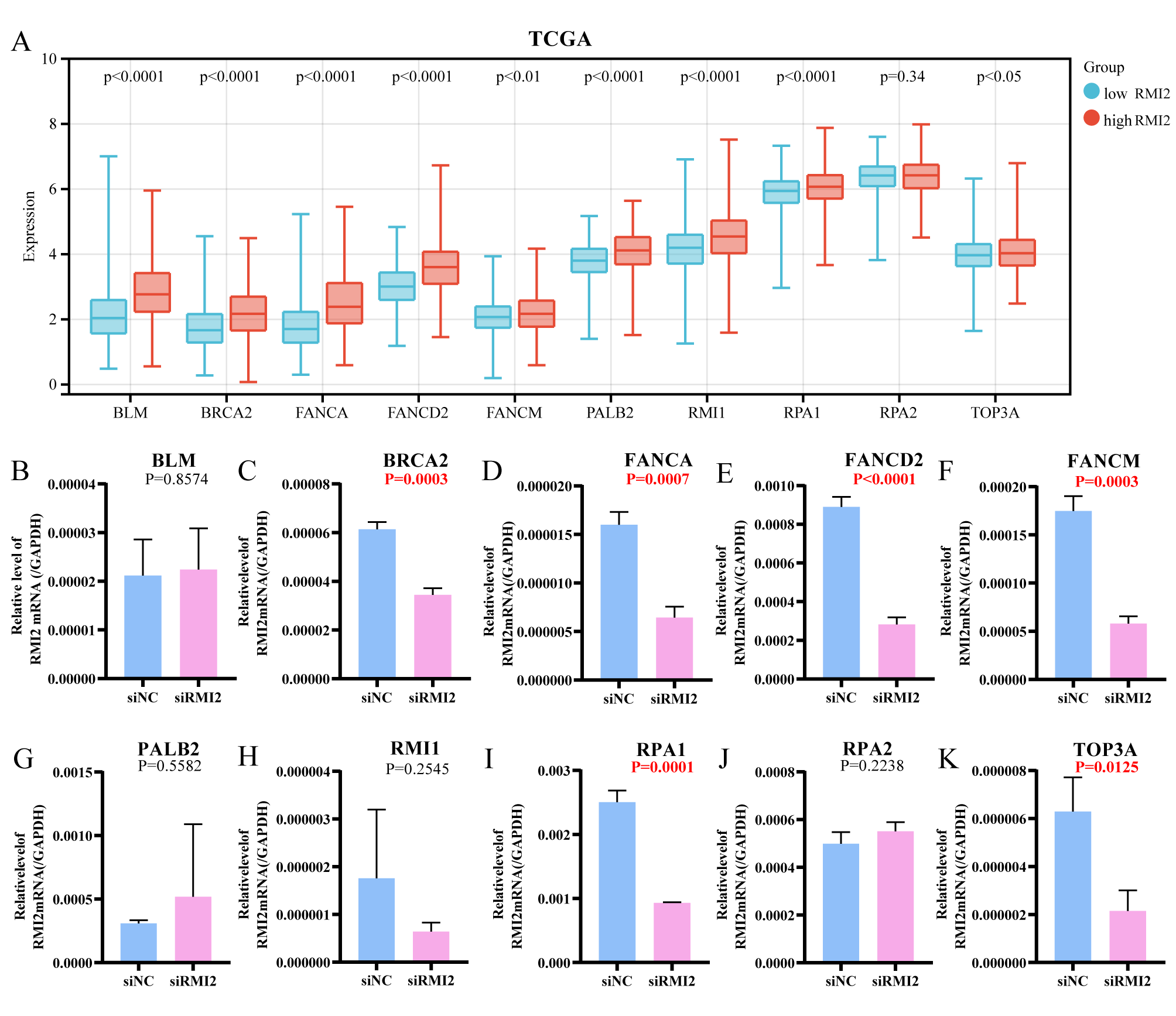

Supplement: Supplementary file 1 — Data S1. [file CAM4-12-8331-s001.zip › CAM4_5533_LijiezhangFigureS3.tif]
